# Synthesis and structure of 1-{(*E*)-[(6-meth­oxy­pyridin-2-yl)methyl­idene]amino}-3-phenyl­thio­urea

**DOI:** 10.1107/S2056989026004111

**Published:** 2026-05-15

**Authors:** Liji Muthirakalayil Abraham, Amirthalingam Arunkumar, Marappan Velusamy, Thangaraja Chinnathangavel, Venugopal Rajendiran

**Affiliations:** a Department of Chemistry School of Basic and Applied Sciences Central University of Tamil Nadu Thiruvarur 610 005 India; b Department of Chemistry North Eastern Hill University,Shillong 793022 India; c Department of Chemistry, Anna University Regional Campus, Madurai 625019, India; University of Aberdeen, United Kingdom

**Keywords:** Schiff base, thio­semicarbazone, crystal structure, Hirshfeld surface analysis

## Abstract

The title com­pound, C_14_H_14_N_4_OS, was crystallized at −20 °C by ether diffusion into an ethanol solution. The dihedral angle between the planes of the aromatic rings is 38.19 (19)° and the mol­ecular conformation is supported by two intra­molecular N—H⋯N hy­dro­gen bonds. In the extended structure, N—H⋯O hy­dro­gen bonds link the mol­ecules into [011] chains. Hirshfeld surface analysis shows that H⋯H (44%), C⋯H/H⋯C (25%) and S⋯H/H⋯S (11.4%) contacts contibute the largest amounts.

## Abstract 2

In the title com­pound, the mol­ecular conformation is supported by two intra­molecular N—H⋯N hy­dro­gen bonds. In the extended structure, N—H⋯O hy­dro­gen bonds link the mol­ecules into [011] chains.

## 1. Chemical context

Thio­semicarbazones, containing an –NH–(C=S)–NH=N– unit, are widely studied members of the Schiff base family: numerous articles discuss various synthetic strategies to pre­pare them and their applications, including optical devices, dyes, pharmaceuticals, biological activity, nanotechnology and catalytic activity (*e.g.* Nidhi *et al.*, 2025 ▸; Kumar *et al.*, 2022 ▸; Shahzad Munawar *et al.*, 2018 ▸). The addition of a pyridin-2-yl moiety to these thio­semicarbazide frameworks can make them more effective in biological applications, such as anti­cancer, anti­microbial, anti­fungal, anti­viral and anti­bacterial activities (Zaware *et al.*, 2025 ▸).

Triapine (3-amino­pyridine-2-carboxaldehyde thio­semicar­bazone; C_7_H_9_N_5_S), an α-*N*-pyridyl­thio­semicarbazone, is a potent ribonucleotide reductase (RR) inhibitor currently undergoing clinical trials (Finch *et al.*, 1999 ▸), which is believed to function by chelating iron ions. COTI-2 is a third-generation thio­semicarbazone that has received FDA (US Food and Drug Administration) orphan drug designation for ovarian cancer. It is undergoing phase 1 clinical trials (Lindemann *et al.*, 2019 ▸). Several metal–Schiff base com­plexes are being studied for their various biological activities (Bajaj *et al.*, 2021 ▸; Pervaiz *et al.*, 2024 ▸; Jiang *et al.*, 2024 ▸; Karpagam *et al.*, 2022 ▸).
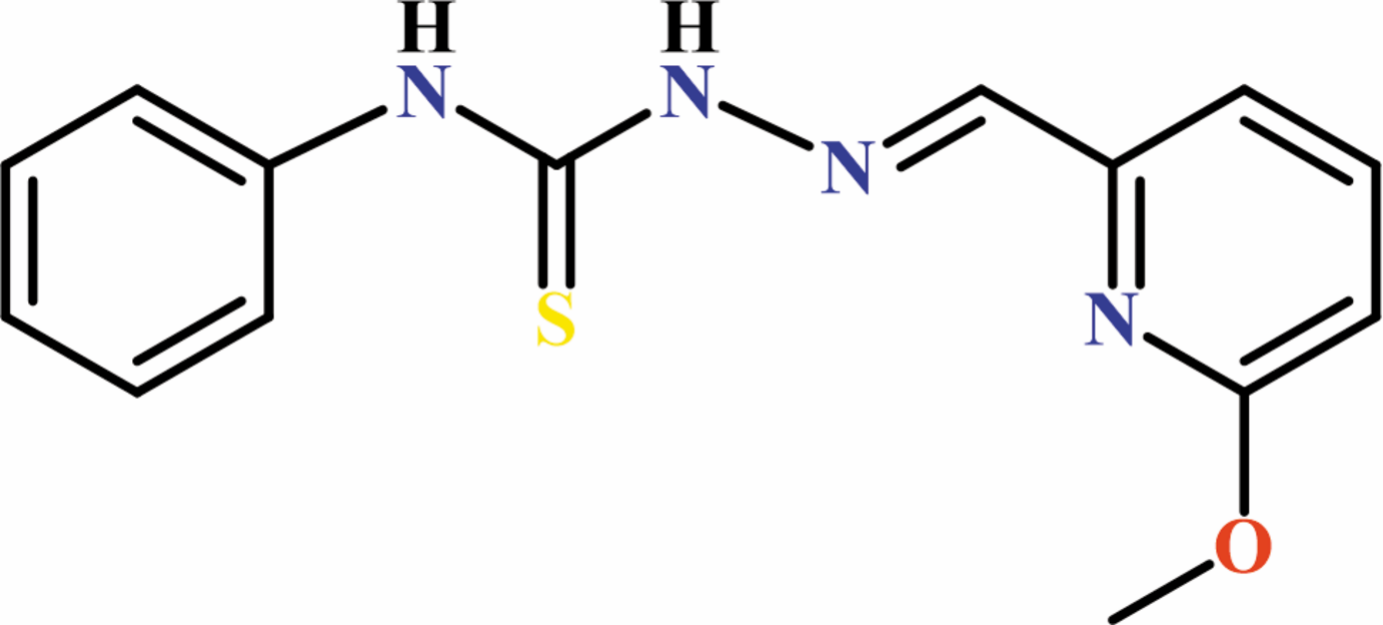


As part of our work in this area, we now describe the syn­thesis, structure and Hirshfeld surface of the title thio­semicarbazone, 1-{(*E*)-[(6-meth­oxy­pyridin-2-yl)methyl­idene]amino}-3-phenyl­thio­urea (**I**), formed from the condensation reaction of 6-meth­oxy­pyridine-2-carboxaldehyde and 4-phenyl thio­semicarbazide.

## 2. Structural commentary

**Figure 1:**
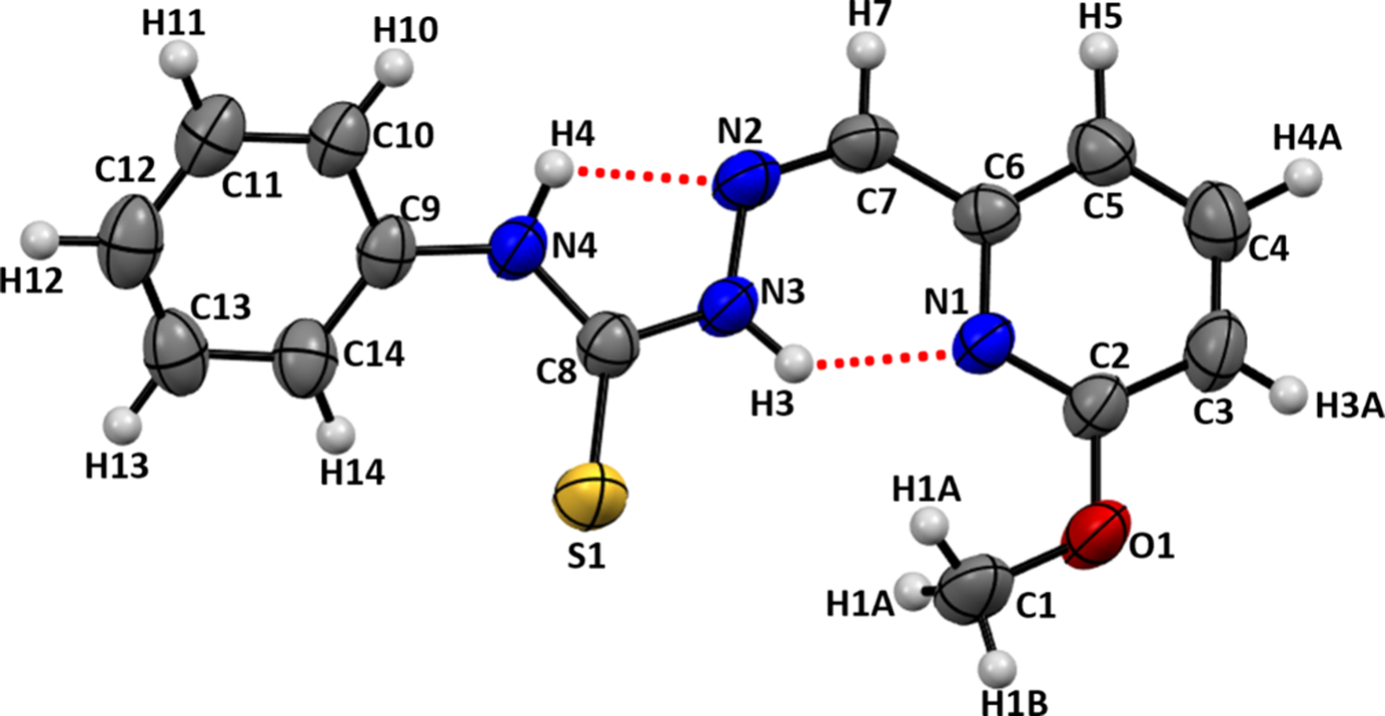
The mol­ecular structure of (**I**), with displacement ellipsoids drawn at the 50% probability level. Intra­molecular N—H⋯N hy­dro­gen bonds are shown as red dashed lines.

Compound (**I**) consists of an *o*-substituted pyrid­yl–Schiff base moiety connected to the phenyl-substituted thio­semicarbazide unit through an azomethine bond. It crystallizes in the ortho­rhom­bic space group *Pna*2_1_, with one mol­ecule in the asymmetric unit, as illustrated in Fig. 1 ▸. The com­pound contains three main structural fragments, a meth­oxy-substituted pyridine ring, an imine linkage and a phenyl­thio­semicarbazide group. The imine bond, with a C7=N2 bond length of 1.287 (5) Å, adopts the expected *E* configuration for the carbon–nitro­gen bond, where the pyridyl ring is placed *trans* to the thio­semicarbazide N atom. The central chain adopts an extended conformation that allows partial electronic delocalization across the pyridine—C=N—N—C(=S)—NH—phenyl framework, as discussed previously for aryl-containing thio­semicarbazones (Palenik *et al.*, 1974 ▸). The N3—N2 bond length in the thio­semicarbazide part is 1.360 (4) Å, which is shorter than the expected N—N single bond length (1.45 Å). Also, the C6—C7 bond length is 1.461 (5) Å, which is shorter than the usual C—C single bond length (1.54 Å), which suggests a partial double-bond character due to the conjugation with the imine group (Bhatia *et al.*, 1977 ▸). Conversely, the thio­carbonyl bond, represented by C8—S1, shows a bond length of 1.658 (4) Å, which is longer than a typical C=S double bond length (1.56 Å), but much shorter than a typical C—S single bond length of 1.82 Å: this also confirms the extensive conjugation within the system.

The dihedral angle between the C2–C6/N1 and C9–C14 aromatic rings is 38.19 (19)°. The C atom of the meth­oxy group attached to the *ortho* position of the pyridine ring is approximately coplanar with the aromatic ring [displacement = 0.080 (6) Å] and the N1—C2—O1 and C3—C2—O1 bond angles are 118.5 (3) and 117.1 (3)°, respectively. The C2(ar­yl)—O1 bond length is 1.353 (4) Å, whereas the O1—C1(meth­yl) bond length is 1.429 (5) Å: this difference is typical for meth­oxy-substituted aromatic systems (Jones & Shaw, 1992 ▸). Two intra­molecular N—H⋯N hy­dro­gen bonds (Table 1 ▸) help to consolidate the mol­ecular conformation: the N3—H3⋯N1 hy­dro­gen bond closes an *S*(6) ring and the N4—H4⋯N2 hy­dro­gen bond an *S*(5) ring. A short C—H⋯S contact is also present.

## 3. Supra­molecular features

**Figure 2:**
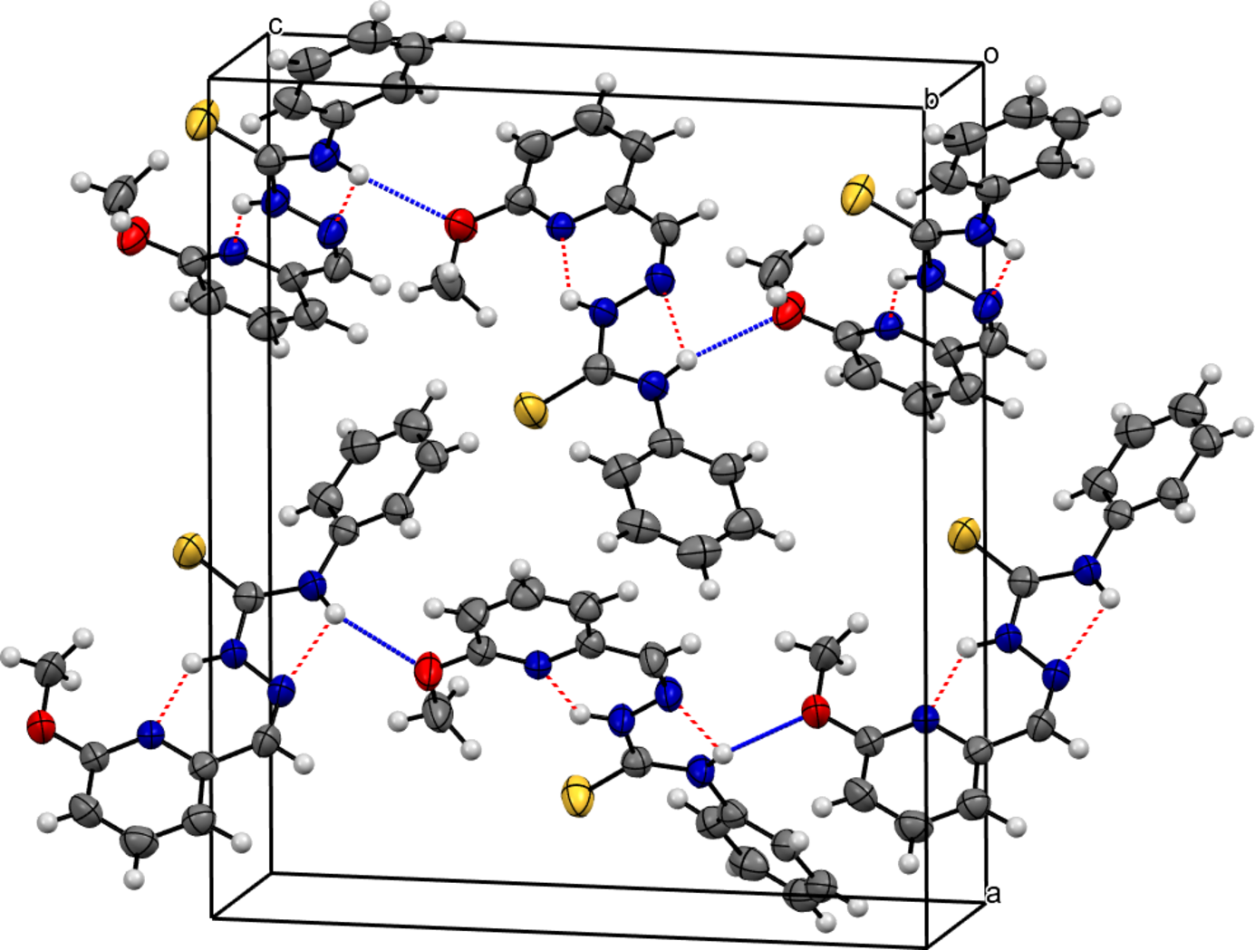
The packing diagram of (**I**), with intra­molecular N—H⋯N hy­dro­gen bonds shown as red dashed lines and inter­molecular N—H⋯O hy­dro­gen bonds shown as blue dashed lines.

In the extended structure, N4—H4⋯O1 hy­dro­gen bonds link the mol­ecules into *C*(10) [01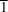] chains (Fig. 2 ▸). This N—H group also forms an intra­molecular hy­dro­gen bond (see above) and the bond-angle sum at the H atom is 357°.

## 4. Hirshfeld surface analysis

**Figure 3:**
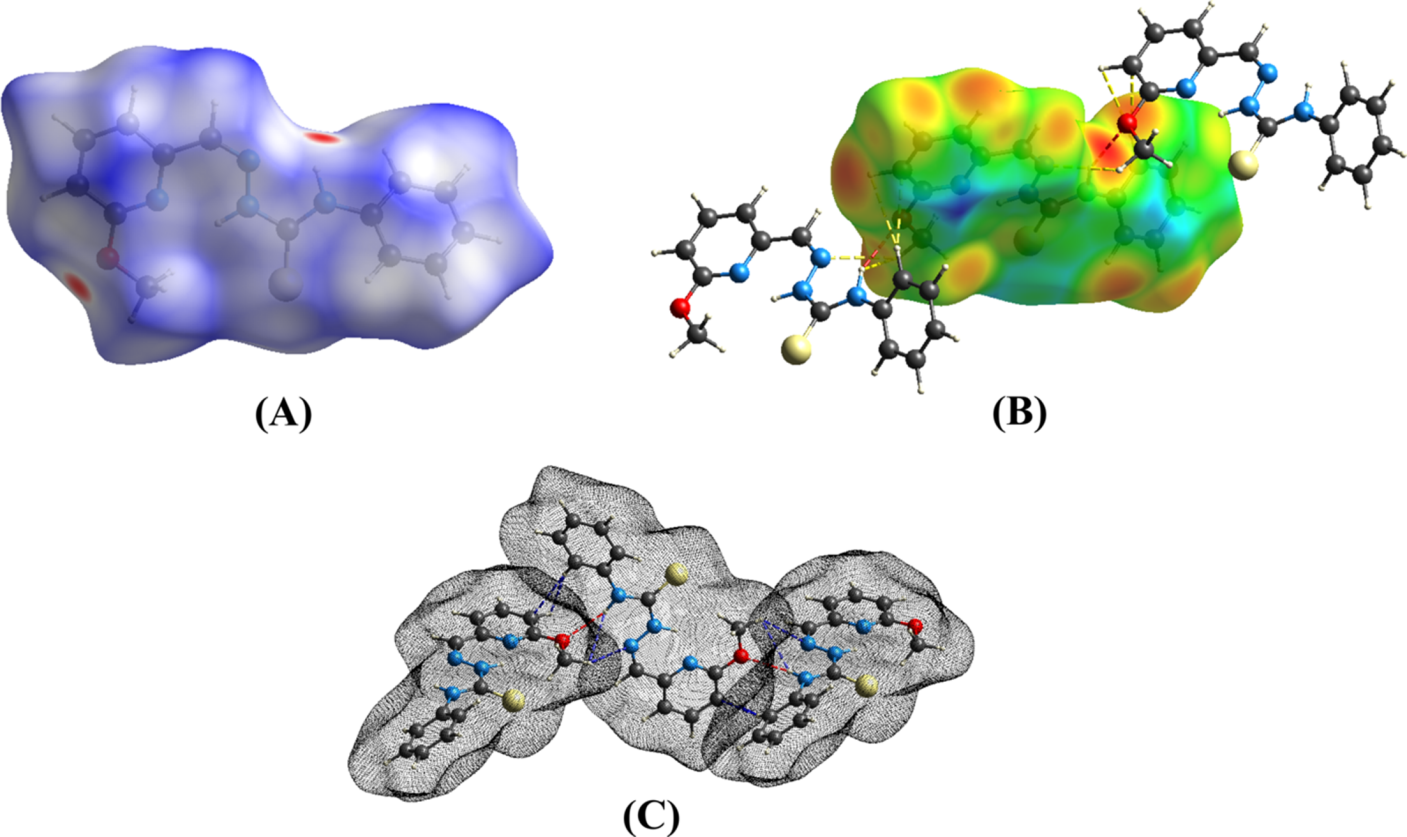
Hirshfeld surfaces of (**I**) mapped over (A) *d*_norm_, (B) *d*_i_ and (C) *d*_norm_ (transparent Hirshfeld surface).

**Figure 4:**
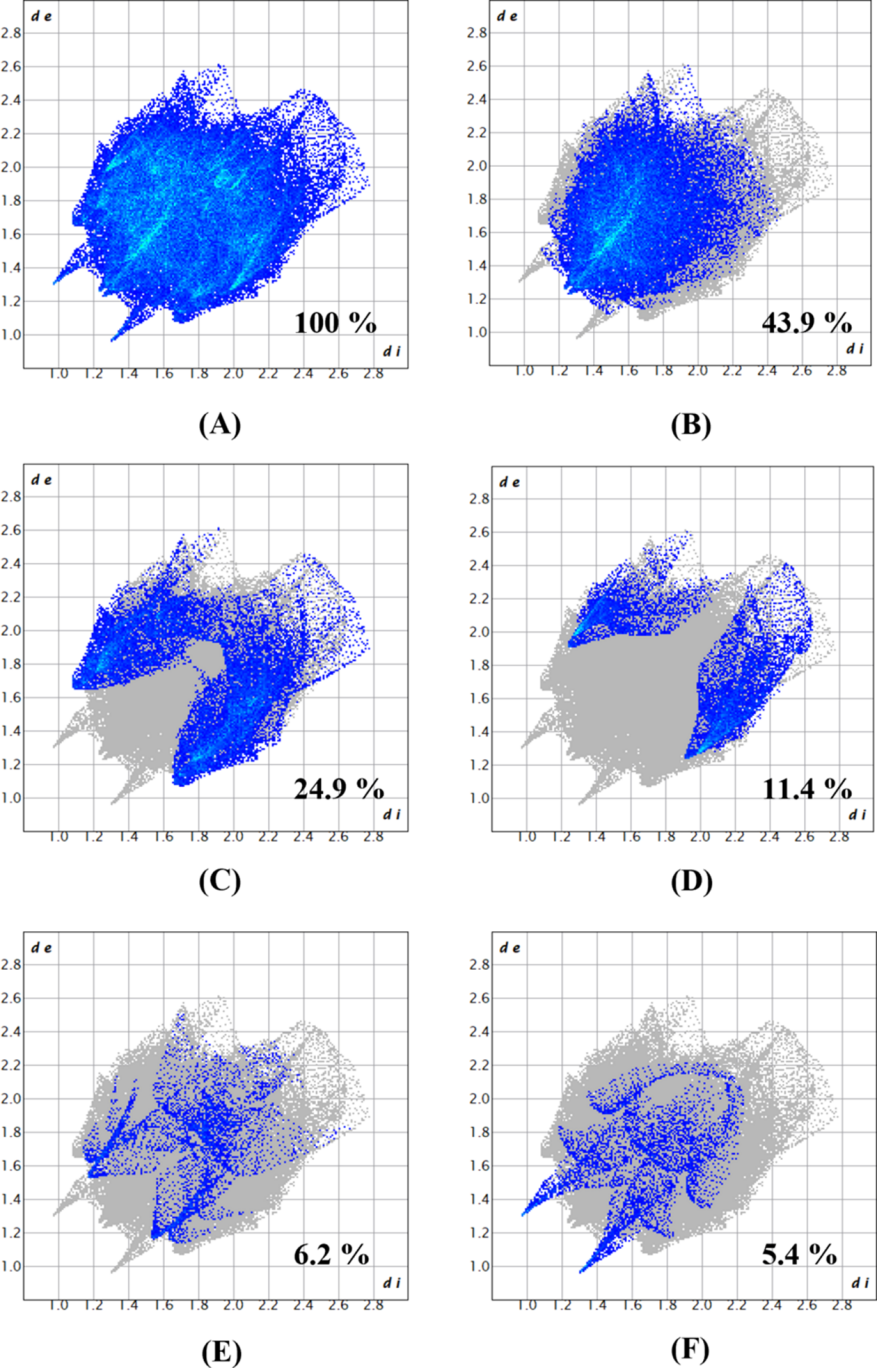
Two-dimensional fingerprint plots for (**I**): (*a*) all inter­actions; (*b*) H⋯H; (*c*) C⋯H/H⋯C; (*d*) S⋯H/H⋯S; (*e*) N⋯H/H⋯N; (*f*) O⋯H/H⋯O.

The Hirshfeld surface analysis of (**I**) was performed using *CrystalExplorer* (Version 21.5; Spackman *et al.*, 2021 ▸) and the *d*_norm_ map is illustrated in Fig. 3 ▸, with the intense red spots corresponding to the donor and acceptor groups for the N—H⋯O hy­dro­gen bond. Two-dimensional fingerprint plots are shown in Fig. 4 ▸. Inter­actions between the H atoms (H⋯H) show the largest contribution (∼44%) to the Hirshfeld surface, with a distance range of *d*_e_ + *d*_i_ ≃ 2.5 Å. Nearly 25% is contributed by C⋯H/H⋯C inter­actions. Similarly, 11.4, 6.2, 5.4, 4.7 and 1.4% of the inter­actions are contributed by S⋯H/H⋯S, N⋯H/H⋯N, O⋯H/H⋯O, C⋯N/N⋯C and S⋯C/C⋯S inter­actions, respectively. Finally, C⋯C, N⋯O/O⋯N and S⋯N/N⋯S inter­actions each contribute less than 1% to the contact surface.

## 5. Database survey

Several thio­semicarbazones have been reported in the literature, but only two structures have a 6-substituted pyridine moiety, *viz.* 6-methyl­pyridine-2-carbaldehyde-*N*(4)-phenyl­thio­semicarbazone and 6-bromo-2-formyl­pyridine-*N*(4)-phenyl­thio­semicarbazone (CSD refcodes BAFGAS and BAFGEW, respectively; Chumakov *et al.*, 2011 ▸).

## 6. Synthesis and crystallization

To 15 ml of a hot ethano­lic solution of 6-meth­oxy­pyridine-2-carboxaldehyde (0.137 g, 1.00 mmol) with two added drops of glacial acetic acid, 4-phenyl­thio­semicarbazide (0.167 g, 1.00 mmol) dissolved in ethanol was added dropwise, and the mixture was refluxed for 2 h, which, on cooling to room temperature, gave a yellow precipitate. The obtained product was then recrystallised from ethanol solution by ether diffusion at −20 °C to give yellow needles of the title com­pound (yield 72%).

## 7. Refinement

Crystal data, data collection and structure refinement details are summarized in Table 2 ▸.

## Supplementary Material

Crystal structure: contains datablock(s) I. DOI: 10.1107/S2056989026004111/hb8209sup1.cif

Structure factors: contains datablock(s) I. DOI: 10.1107/S2056989026004111/hb8209Isup2.hkl

Supporting information file. DOI: 10.1107/S2056989026004111/hb8209Isup3.cml

CCDC reference: 2535197

Additional supporting information:  crystallographic information; 3D view; checkCIF report

## Figures and Tables

**Table 1:** Hydrogen-bond geometry (Å, °)

*D*—H⋯*A*	*D*—H	H⋯*A*	*D*⋯*A*	*D*—H⋯*A*
N3—H3⋯N1	0.89 (3)	1.99 (4)	2.662 (4)	131 (3)
N4—H4⋯N2	0.91 (4)	2.07 (5)	2.576 (5)	114 (4)
N4—H4⋯O1^i^	0.91 (4)	2.38 (5)	3.230 (4)	155 (3)
C14—H14⋯S1	0.97 (3)	2.78 (3)	3.249 (5)	110 (2)

Symmetry code: (i) 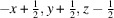.

**Table 2:** Experimental details

Crystal data	
Chemical formula	C_14_H_14_N_4_OS
*M*_r_	286.35
Crystal system, space group	Orthorhombic, *Pna*2_1_
Temperature (K)	296
*a*, *b*, *c* (Å)	17.811 (4), 5.2400 (14), 15.340 (4)
*V* (Å^3^)	1431.7 (6)
*Z*	4
Radiation type	Mo *K*α
μ (mm^−1^)	0.23
Crystal size (mm)	0.42 × 0.37 × 0.26

Data collection	
Diffractometer	Bruker
No. of measured, independent and observed [*I* > 2σ(*I*)] reflections	23242, 3409, 2258
*R*_int_	0.078
(sin θ/λ)_max_ (Å^−1^)	0.660

Refinement	
*R*[*F*^2^ > 2σ(*F*^2^)], *wR*(*F*^2^), *S*	0.040, 0.096, 1.02
No. of reflections	3409
No. of parameters	238
No. of restraints	1
H-atom treatment	All H-atom parameters refined
Δρ_max_, Δρ_min_ (e Å^−3^)	0.17, −0.16
Absolute structure	Flack *x* determined using 859 quotients [(*I*^+^−(*I*^−^)]/[(*I*^+^)+(*I*^−^)] (Parsons *et al.*, 2013 ▸)
Absolute structure parameter	−0.04 (6)

Computer programs: *APEX3* and *SAINT* (Bruker, 2018 ▸), *SHELXT2014* (Sheldrick, 2015*a* ▸), *SHELXL2018* (Sheldrick, 2015*b* ▸), *Mercury* (Macrae *et al.*, 2020 ▸) and *publCIF* (Westrip, 2010 ▸).

## supplementary crystallographic information

### 1-{(E)-[(6-Methoxypyridin-2-yl)methylidene]amino}-3-phenylthiourea Crystal data

**Table:**

C_14_H_14_N_4_OS	*D*_x_ = 1.328 Mg m^−^^3^
*M**_r_* = 286.35	Mo *K*α radiation, λ = 0.71073 Å
Orthorhombic, *P**n**a*2_1_	Cell parameters from 3409 reflections
*a* = 17.811 (4) Å	θ = 2.3–28.0°
*b* = 5.2400 (14) Å	µ = 0.23 mm^−^^1^
*c* = 15.340 (4) Å	*T* = 296 K
*V* = 1431.7 (6) Å^3^	Needle, yellow
*Z* = 4	0.42 × 0.37 × 0.26 mm
*F*(000) = 600	

### 1-{(E)-[(6-Methoxypyridin-2-yl)methylidene]amino}-3-phenylthiourea Data collection

**Table:**

Bruker diffractometer	*R*_int_ = 0.078
ω and phi scans	θ_max_ = 28.0°, θ_min_ = 2.3°
23242 measured reflections	*h* = −23→23
3409 independent reflections	*k* = −6→6
2258 reflections with *I* > 2σ(*I*)	*l* = −20→20

### 1-{(E)-[(6-Methoxypyridin-2-yl)methylidene]amino}-3-phenylthiourea Refinement

**Table:**

Refinement on *F*^2^	All H-atom parameters refined
Least-squares matrix: full	*w* = 1/[σ^2^(*F*_o_^2^) + (0.0348*P*)^2^ + 0.1169*P*] where *P* = (*F*_o_^2^ + 2*F*_c_^2^)/3
*R*[*F*^2^ > 2σ(*F*^2^)] = 0.040	(Δ/σ)_max_ < 0.001
*wR*(*F*^2^) = 0.096	Δρ_max_ = 0.17 e Å^−^^3^
*S* = 1.02	Δρ_min_ = −0.16 e Å^−^^3^
3409 reflections	Extinction correction: SHELXL2018 (Sheldrick, 2015*b*), Fc^*^=kFc[1+0.001xFc^2^λ^3^/sin(2θ)]^-1/4^
238 parameters	Extinction coefficient: 0.030 (3)
1 restraint	Absolute structure: Flack *x* determined using 859 quotients [(I+)-(I-)]/[(I+)+(I-)] (Parsons *et al.*, 2013)
Primary atom site location: dual	Absolute structure parameter: −0.04 (6)
Hydrogen site location: difference Fourier map	

### 1-{(E)-[(6-Methoxypyridin-2-yl)methylidene]amino}-3-phenylthiourea Special details

**Table:**

Geometry. All esds (except the esd in the dihedral angle between two l.s. planes) are estimated using the full covariance matrix. The cell esds are taken into account individually in the estimation of esds in distances, angles and torsion angles; correlations between esds in cell parameters are only used when they are defined by crystal symmetry. An approximate (isotropic) treatment of cell esds is used for estimating esds involving l.s. planes.

### 1-{(E)-[(6-Methoxypyridin-2-yl)methylidene]amino}-3-phenylthiourea Fractional atomic coordinates and isotropic or equivalent isotropic displacement parameters (Å^2^)

**Table:**

	*x*	*y*	*z*	*U*_iso_*/*U*_eq_	
S1	0.40063 (6)	0.6279 (2)	0.58169 (8)	0.0694 (4)	
N1	0.21315 (14)	0.0452 (5)	0.58787 (19)	0.0431 (6)	
N3	0.29424 (17)	0.3856 (6)	0.4999 (2)	0.0479 (7)	
O1	0.22148 (15)	−0.0365 (6)	0.73431 (15)	0.0662 (9)	
N2	0.25657 (17)	0.3109 (7)	0.4272 (2)	0.0538 (8)	
N4	0.36505 (17)	0.6321 (6)	0.4102 (2)	0.0483 (7)	
C2	0.1914 (2)	−0.0897 (7)	0.6554 (2)	0.0486 (9)	
C9	0.4233 (2)	0.7937 (7)	0.3791 (2)	0.0454 (8)	
C8	0.3525 (2)	0.5529 (6)	0.4928 (2)	0.0463 (8)	
C6	0.1817 (2)	−0.0119 (7)	0.5095 (2)	0.0439 (8)	
C10	0.4548 (2)	0.7346 (8)	0.2986 (3)	0.0512 (9)	
C11	0.5105 (2)	0.8881 (9)	0.2637 (3)	0.0585 (11)	
C7	0.2064 (2)	0.1351 (8)	0.4338 (2)	0.0534 (9)	
C3	0.1383 (2)	−0.2814 (9)	0.6519 (3)	0.0628 (11)	
C14	0.4467 (2)	1.0090 (8)	0.4234 (3)	0.0547 (9)	
C5	0.1280 (2)	−0.2000 (7)	0.5005 (3)	0.0531 (9)	
C13	0.5027 (2)	1.1614 (8)	0.3869 (3)	0.0607 (11)	
C12	0.5341 (3)	1.0994 (9)	0.3078 (3)	0.0654 (12)	
C4	0.1065 (2)	−0.3363 (7)	0.5728 (4)	0.0621 (11)	
C1	0.2800 (3)	0.1498 (13)	0.7369 (3)	0.0737 (14)	
H10	0.441 (2)	0.584 (8)	0.269 (3)	0.067 (12)*	
H4A	0.070 (2)	−0.481 (9)	0.568 (3)	0.085 (14)*	
H13	0.515 (2)	1.312 (8)	0.418 (3)	0.066 (12)*	
H14	0.423 (2)	1.057 (6)	0.478 (2)	0.047 (10)*	
H3A	0.127 (2)	−0.373 (7)	0.698 (3)	0.059 (12)*	
H5	0.105 (2)	−0.236 (8)	0.438 (3)	0.068 (12)*	
H7	0.183 (2)	0.093 (7)	0.378 (3)	0.061 (11)*	
H3	0.285 (2)	0.316 (7)	0.552 (2)	0.057 (11)*	
H12	0.572 (3)	1.212 (8)	0.284 (3)	0.080 (13)*	
H11	0.531 (3)	0.848 (8)	0.209 (4)	0.095 (17)*	
H4	0.338 (3)	0.540 (7)	0.371 (3)	0.071 (13)*	
H1A	0.321 (3)	0.107 (8)	0.687 (4)	0.096 (15)*	
H1B	0.293 (3)	0.145 (8)	0.792 (3)	0.081 (15)*	
H1C	0.258 (3)	0.319 (11)	0.722 (4)	0.096 (19)*	

### 1-{(E)-[(6-Methoxypyridin-2-yl)methylidene]amino}-3-phenylthiourea Atomic displacement parameters (Å^2^)

**Table:**

	*U*^11^	*U*^22^	*U*^33^	*U*^12^	*U*^13^	*U*^23^
S1	0.0726 (7)	0.0868 (7)	0.0489 (5)	−0.0254 (6)	−0.0119 (6)	0.0070 (6)
N1	0.0374 (14)	0.0560 (16)	0.0357 (13)	−0.0022 (12)	−0.0006 (14)	0.0066 (15)
N3	0.0458 (19)	0.0634 (18)	0.0344 (15)	−0.0129 (14)	−0.0020 (13)	0.0081 (15)
O1	0.0607 (19)	0.097 (2)	0.0404 (14)	−0.0231 (16)	−0.0035 (13)	0.0160 (15)
N2	0.0535 (19)	0.073 (2)	0.0353 (16)	−0.0123 (17)	−0.0057 (14)	0.0069 (15)
N4	0.0474 (18)	0.0577 (19)	0.0397 (16)	−0.0103 (15)	−0.0020 (13)	0.0102 (15)
C2	0.042 (2)	0.061 (2)	0.042 (2)	−0.0033 (17)	0.0007 (17)	0.0089 (18)
C9	0.0398 (17)	0.045 (2)	0.051 (2)	0.0035 (16)	−0.0027 (16)	0.0145 (16)
C8	0.045 (2)	0.0474 (19)	0.0462 (19)	−0.0006 (17)	−0.0011 (16)	0.0043 (17)
C6	0.0400 (18)	0.049 (2)	0.0422 (18)	0.0011 (16)	−0.0021 (16)	−0.0016 (15)
C10	0.047 (2)	0.055 (2)	0.052 (2)	0.0010 (19)	0.0019 (18)	0.0135 (19)
C11	0.049 (2)	0.069 (3)	0.058 (2)	0.005 (2)	0.005 (2)	0.019 (2)
C7	0.056 (2)	0.067 (2)	0.0366 (18)	−0.010 (2)	−0.0080 (17)	0.0019 (18)
C3	0.056 (2)	0.070 (3)	0.062 (3)	−0.013 (2)	0.007 (2)	0.018 (2)
C14	0.054 (2)	0.046 (2)	0.064 (3)	0.0043 (18)	0.006 (2)	0.0062 (19)
C5	0.049 (2)	0.056 (2)	0.054 (2)	−0.0049 (19)	−0.0026 (18)	−0.003 (2)
C13	0.054 (2)	0.046 (2)	0.083 (3)	−0.0013 (19)	−0.009 (2)	0.012 (2)
C12	0.051 (3)	0.064 (3)	0.082 (3)	0.002 (2)	0.001 (2)	0.026 (3)
C4	0.053 (2)	0.060 (2)	0.073 (3)	−0.0134 (19)	−0.001 (2)	0.005 (2)
C1	0.068 (3)	0.108 (4)	0.045 (2)	−0.025 (3)	−0.015 (2)	0.013 (3)

### 1-{(E)-[(6-Methoxypyridin-2-yl)methylidene]amino}-3-phenylthiourea Geometric parameters (Å, º)

**Table:**

S1—C8	1.658 (4)	C10—H10	0.94 (4)
N1—C2	1.312 (4)	C11—C12	1.364 (6)
N1—C6	1.360 (4)	C11—H11	0.93 (5)
N3—N2	1.360 (4)	C7—H7	0.97 (4)
N3—C8	1.363 (4)	C3—C4	1.369 (7)
N3—H3	0.89 (4)	C3—H3A	0.88 (4)
O1—C2	1.354 (4)	C14—C13	1.396 (6)
O1—C1	1.429 (5)	C14—H14	0.98 (4)
N2—C7	1.287 (5)	C5—C4	1.374 (7)
N4—C8	1.352 (4)	C5—H5	1.07 (4)
N4—C9	1.422 (5)	C13—C12	1.374 (6)
N4—H4	0.91 (5)	C13—H13	0.95 (4)
C2—C3	1.381 (5)	C12—H12	0.97 (5)
C9—C14	1.382 (6)	C4—H4A	1.00 (5)
C9—C10	1.390 (5)	C1—H1A	1.09 (5)
C6—C5	1.381 (5)	C1—H1B	0.88 (5)
C6—C7	1.461 (5)	C1—H1C	1.00 (6)
C10—C11	1.386 (6)		
			
C2—N1—C6	117.2 (3)	N2—C7—C6	130.5 (3)
N2—N3—C8	119.7 (3)	N2—C7—H7	113 (2)
N2—N3—H3	122 (2)	C6—C7—H7	116 (2)
C8—N3—H3	118 (2)	C4—C3—C2	118.0 (4)
C2—O1—C1	117.1 (3)	C4—C3—H3A	120 (3)
C7—N2—N3	118.9 (3)	C2—C3—H3A	121 (3)
C8—N4—C9	128.1 (3)	C9—C14—C13	119.0 (4)
C8—N4—H4	112 (3)	C9—C14—H14	120 (2)
C9—N4—H4	119 (3)	C13—C14—H14	121 (2)
N1—C2—O1	118.5 (3)	C4—C5—C6	118.9 (4)
N1—C2—C3	124.4 (4)	C4—C5—H5	122 (2)
O1—C2—C3	117.1 (3)	C6—C5—H5	119 (2)
C14—C9—C10	119.8 (4)	C12—C13—C14	120.7 (4)
C14—C9—N4	122.7 (4)	C12—C13—H13	124 (3)
C10—C9—N4	117.3 (3)	C14—C13—H13	116 (3)
N4—C8—N3	113.5 (3)	C11—C12—C13	120.3 (4)
N4—C8—S1	127.8 (3)	C11—C12—H12	121 (3)
N3—C8—S1	118.7 (3)	C13—C12—H12	118 (3)
N1—C6—C5	122.1 (3)	C3—C4—C5	119.4 (4)
N1—C6—C7	117.5 (3)	C3—C4—H4A	120 (3)
C5—C6—C7	120.4 (4)	C5—C4—H4A	121 (3)
C11—C10—C9	120.2 (4)	O1—C1—H1A	109 (3)
C11—C10—H10	119 (3)	O1—C1—H1B	102 (3)
C9—C10—H10	121 (3)	H1A—C1—H1B	119 (4)
C12—C11—C10	120.0 (4)	O1—C1—H1C	108 (3)
C12—C11—H11	120 (3)	H1A—C1—H1C	107 (4)
C10—C11—H11	120 (3)	H1B—C1—H1C	111 (5)
			
C8—N3—N2—C7	173.5 (4)	C9—C10—C11—C12	−0.7 (6)
C6—N1—C2—O1	−179.8 (3)	N3—N2—C7—C6	−1.3 (7)
C6—N1—C2—C3	−1.1 (5)	N1—C6—C7—N2	2.7 (7)
C1—O1—C2—N1	−5.3 (6)	C5—C6—C7—N2	−177.7 (4)
C1—O1—C2—C3	175.9 (4)	N1—C2—C3—C4	0.6 (6)
C8—N4—C9—C14	−41.6 (6)	O1—C2—C3—C4	179.3 (4)
C8—N4—C9—C10	141.5 (4)	C10—C9—C14—C13	−1.1 (5)
C9—N4—C8—N3	−176.2 (3)	N4—C9—C14—C13	−178.0 (3)
C9—N4—C8—S1	1.6 (6)	N1—C6—C5—C4	−1.0 (6)
N2—N3—C8—N4	1.5 (5)	C7—C6—C5—C4	179.4 (3)
N2—N3—C8—S1	−176.5 (3)	C9—C14—C13—C12	0.3 (6)
C2—N1—C6—C5	1.3 (5)	C10—C11—C12—C13	−0.2 (6)
C2—N1—C6—C7	−179.2 (3)	C14—C13—C12—C11	0.4 (6)
C14—C9—C10—C11	1.3 (5)	C2—C3—C4—C5	−0.3 (6)
N4—C9—C10—C11	178.4 (3)	C6—C5—C4—C3	0.5 (6)

### 1-{(E)-[(6-Methoxypyridin-2-yl)methylidene]amino}-3-phenylthiourea Hydrogen-bond geometry (Å, º)

**Table:**

*D*—H···*A*	*D*—H	H···*A*	*D*···*A*	*D*—H···*A*
N3—H3···N1	0.89 (3)	1.99 (4)	2.662 (4)	131 (3)
N4—H4···N2	0.91 (4)	2.07 (5)	2.576 (5)	114 (4)
N4—H4···O1^i^	0.91 (4)	2.38 (5)	3.230 (4)	155 (3)
C14—H14···S1	0.97 (3)	2.78 (3)	3.249 (5)	110 (2)

Symmetry code: (i) −*x*+1/2, *y*+1/2, *z*−1/2.
